# Catalytic Profile of *Arabidopsis* Peroxidases, AtPrx-2, 25 and 71, Contributing to Stem Lignification

**DOI:** 10.1371/journal.pone.0105332

**Published:** 2014-08-19

**Authors:** Jun Shigeto, Mariko Nagano, Koki Fujita, Yuji Tsutsumi

**Affiliations:** Faculty of Agriculture, Kyushu University, Fukuoka, Japan; National Taiwan University, Taiwan

## Abstract

Lignins are aromatic heteropolymers that arise from oxidative coupling of lignin precursors, including lignin monomers (*p*-coumaryl, coniferyl, and sinapyl alcohols), oligomers, and polymers. Whereas plant peroxidases have been shown to catalyze oxidative coupling of monolignols, the oxidation activity of well-studied plant peroxidases, such as horseradish peroxidase C (HRP-C) and AtPrx53, are quite low for sinapyl alcohol. This characteristic difference has led to controversy regarding the oxidation mechanism of sinapyl alcohol and lignin oligomers and polymers by plant peroxidases. The present study explored the oxidation activities of three plant peroxidases, AtPrx2, AtPrx25, and AtPrx71, which have been already shown to be involved in lignification in the *Arabidopsis* stem. Recombinant proteins of these peroxidases (rAtPrxs) were produced in *Escherichia coli* as inclusion bodies and successfully refolded to yield their active forms. rAtPrx2, rAtPrx25, and rAtPrx71 were found to oxidize two syringyl compounds (2,6-dimethoxyphenol and syringaldazine), which were employed here as model monolignol compounds, with higher specific activities than HRP-C and rAtPrx53. Interestingly, rAtPrx2 and rAtPrx71 oxidized syringyl compounds more efficiently than guaiacol. Moreover, assays with ferrocytochrome *c* as a substrate showed that AtPrx2, AtPrx25, and AtPrx71 possessed the ability to oxidize large molecules. This characteristic may originate in a protein radical. These results suggest that the plant peroxidases responsible for lignin polymerization are able to directly oxidize all lignin precursors.

## Introduction

Lignin is a main component of vascular plant cell walls and possesses a complex and irregular structure. In angiosperms, lignins consist mainly of two monolignols, coniferyl (4-hydroxy-3-methoxycinnamyl) and sinapyl (3,5-dimethoxy-4-hydroxycinnamyl) alcohols, which polymerize through at least five different linkage types and result in 4-hydroxy-3-methoxyphenyl (guaiacyl, G) and 3,5-dimethoxy-4-hydroxyphenyl (syringyl, S) units, respectively. Monolignols are supplied to the cell wall and polymerized to fill, together with hemicellulose, the spaces between cellulose microfibrils; this polymerization proceeds through oxidative coupling catalyzed by plant peroxidases [1]. Based on the “End-wise” polymerization process, monolignol radicals can be coupled to a growing lignin polymer to produce a lignin macromolecule [2].

Plant peroxidases, which include large numbers of isoforms, participate in a broad range of physiological processes besides lignification, including suberin formation, phytoalexins synthesis, metabolism of reactive oxygen and nitrogen species, and programmed cell death [3]. To date, there is limited information available regarding the role of individual isoforms. Their contribution to lignification have been evaluated in several studies that have demonstrated that the up- or down-regulation of a target peroxidase gene is an effective strategy. For example, overexpression of a basic peroxidase in tomato leads to an increase in lignin content [4], and suppression of PrxA3a in aspen decreases lignin content [5]. Transgenic tobacco suppressed TP60 causes great decreases (up to 50%) in lignin content [6] and xylem with both fibers and vessels having thin cell walls [7]. Studies designed to identify plant peroxidases that contribute to lignification have also employed other approaches, such as enzyme purification using the enzyme's oxidation abilities toward monolignols and lignin polymers as an index. It has been reported that some plant peroxidases could oxidize sinapyl alcohol so far [8]. However, only cationic cell wall-bound peroxidase (CWPO-C), a peroxidase isozyme from *Populus alba* L. (poplar) cell wall, has been verified to serve oxidation of monolignols and lignin polymer [9], [10]. Previously, this research group has focused on seven *Arabidopsis* plant peroxidases selected using amino acid similarities to CWPO-C as the probe and found that AtPrx2 or AtPrx25 deficiency led both decreased total lignin content and altered lignin structure, including cell wall thinning in the stem. In addition, AtPrx71 deficiency led an altered stem lignin structure, although the lignin content is not decreased [11]. These results provided *in vivo* evidence that AtPrx-2, 25, and 71 are involved in *Arabidopsis* stem lignification.

On the other hand, the catalytic mechanism of lignin polymerization by plant peroxidases, including the above three peroxidases, toward monolignols and growing lignin polymers is still being discussed. Because of the lack of oxidation activities toward lignin polymers and sinapyl alcohol, well-studied plant peroxidases, such as horseradish peroxidase C (HRP-C) and AtPrx53, are not matched as lignin polymerization enzymes. CWPO-C's unique oxidation ability does qualify as such an enzyme, and its activity provided by two protein surface tyrosine residues (Tyr74 and Tyr177) that can form a radical which is then available as an oxidation active site [12], [13]. The biochemical characterization of CWPO-C *in vitro* has clarified that it can catalyze lignin polymerization without suffering steric hindrance owing to the substrate molecular size by conducting a one-electron oxidation reaction on monolignols and lignin oligomers and polymers on the protein surface [12]. Although CWPO-C's substrate oxidation system allows explanation of lignin polymerization catalyzed by plant peroxidases, further studies are required to reveal CWPO-C's physiological function. AtPrx-2, 25, and 71 are attractive for characterization of their oxidation activities because they have high amino acid similarity to CWPO-C and have already been shown to be responsible for lignification. AtPrx2 conserves its Tyr78 corresponding to catalytic Tyr74 of CWPO-C and shares 44% amino acid identity with CWPO-C. AtPrx25, with 64% amino acid identity with CWPO-C, is the only peroxidase that conserves its Tyr177 in *Arabidopsis*. And AtPrx71 possesses the highest amino acid sequence identity (68%) with CWPO-C. It would, therefore, be interesting to verify whether their oxidation activities are as effective for lignin polymerization as is CWPO-C. This study focused on three lignification related peroxidases, AtPrx-2, 25 and 71, in *Arabidopsis*, and the ability of their recombinant proteins to oxidize monolignols model compounds and large molecule were characterized *in vitro*. The relevance of their oxidation activity is discussed with respect to the common lignin polymerization mechanism catalyzed by peroxidases in vascular plants.

## Materials and Methods

### Plasmid construction and production of rAtPrx inclusion bodies

A cDNA library constructed from whole tissues of *Arabidopsis* (ecotype Columbia) was used as a template for PCR amplification of the targeted genes with KOD-Plus-DNA polymerase (Toyobo Co., Ltd., Osaka, Japan). Gene-specific primers containing *Bam*HI and *Sac*I restriction enzyme sites (Table S1) were used in PCR to amplify the coding sequence for the predicted mature proteins of AtPrx-2, 25, 53, and 71. Subsequent protein construction employing these primers eliminated from the precursor AtPrx proteins the N-terminal amino acids that would constitute the signal-peptide. The amplified fragment was ligated into pBluscript II KS (+) (Stratagene Corp., La Jolla, CA, USA) using restriction site *Bam*HI/*Sac*I and sequenced to ensure that no mutation was introduced during the subcloning process. Every cDNA was then transferred as a *Bam*HI/*Sac*I fragment into pQE-30-Xa to yield pQE-30-Xa-AtPrx. *Escherichia coli* (*E*. *coli*) M15 (pREP4) cells were transformed with pQE-30-Xa-AtPrx and grown in LB medium containing ampicillin and kanamycin (each at 50 µg·mL^−1^) at 37°C and with vigorous shaking. When the *A*_600 nm_ reached 0.2, ITPG was added to a final concentration of 0.4 mM. After further growth for 4 h, bacterial pellets were pelleted by centrifugation at 10 000×g for 10 min and hydrophobic proteins containing inclusion bodies prepared with Bugbuster Protein Extraction Reagent (Novagen, EMD Biosciences, Inc., Darmstadt, Germany) containing benzonase nuclease and lysozyme (25 units·mL^−1^ and 200 µg·mL^−1^, respectively) according to the manufacturer's protocol. The final pellets were stored at −80°C until the day of the refolding experiments.

### Production and purification of recombinant AtPrx and CWPO-C

For each recombinant protein, the composition of the refolding mixture and refolding time were optimized in preliminary experiments (Table 1). After refolding, each refolding mixture of rAtPrx-2, 53, and 71 was dialyzed against 50 mM HEPES-NaOH (pH 7.0), 20 mM Tris-HCl (pH 8.5), and 50 mM MES-NaOH (pH 5.7), respectively. Then, insoluble material was sedimented by centrifugation at 14 000×g for 20 min and the supernatant filtered using cellulose acetate filters with 0.45 µm pore size (Advantec MFS, Inc., Dublin, CA, USA). Renatured rAtPrx-2 and 71 were purified using a cation-exchange chromatography column (Mono Q; GE Healthcare Bio-Sciences UK Ltd., Little Chalfont, UK) connected to a fast protein liquid chromatography system (Amersham Pharmacia Biotech, Inc., Piscataway, NJ, USA) with a linear gradient of 0–1.0 M NaCl in HEPES-NaOH and 20 mM Tris-HCl (pH 7.0 and 8.5, respectively). Renatured rAtPrx53 was purified using an anion-exchange chromatography column (Mono S; GE Healthcare Bio-Sciences UK Ltd.) with a linear gradient of 0–0.1 M NaCl in 20 mM Tris-HCl (pH 8.5). Finally, for renatured rAtPrx25 purification, NaCl was added into a refolding mixture of rAtPrx25 to a final 2.0 M concentration. After centrifugation at 14 000×g for 20 min, the supernatant was loaded onto a hydrophobic interaction chromatography column (HiPrep Butyl FF 16/10; GE Healthcare Bio-Sciences UK Ltd.) and eluted with a NaCl gradient of 2.0–0 M in buffer containing 50 mM Tris-HCl (pH 7.5) and 100 mM CaCl_2_. The resulting enzyme fractions with the highest specific activity were pooled and concentrated using Pierce Protein Concentrators (20 ml/20K MWCO; Thermo Fisher Scientific Inc., Rockford, IL, USA). The concentrated sample (2 ml) was applied onto a HiLoad 16/60 Superdex 75 column (GE Healthcare Bio-Sciences UK Ltd.), and eluted with buffer containing 50 mM Tris-HCl (pH 7.5) and 100 mM CaCl_2_ and 200 mM NaCl at a 0.25 ml·min^−1^ flow rate. Fractions containing renatured rAtPrx with the highest specific activity were used for peroxidase assays. The production and purification procedures of rCWPO-C were the same as previously described [13].

**Table 1:** Refolding conditions of inclusion body proteins expressed in *E. coli*.

	Denaturizing agent (M)	pH	CaCl_2_ (mM)	Hemin (µM)	Oxidizing agent (mM)	Reducing agent (mM)	Protein conc. (mg/ml)	Time (h)	Glycerol (%)
rAtPrx2	Urea, 1.75	11	80	5	GSSG, 0.35	GSH, 0.11	0.1	96	5
rAtPrx25	Guanidine, 0.25	9.5	160	10	Cystine, 0.50	Cystein, 0.50	0.2	24	5
rAtPrx53	Urea, 2.75	9.5	5	15	GSSG, 0.70	GSH, 0.21	0.3	72	5
rAtPrx71	Urea, 3.25	9.5	40	10	GSSG, 0.70	GSH, 0.21	0.2	48	5

### Peroxidase assay

The specific activity of rAtPrx toward guaiacol, 2,6-DMP, and syringaldazine were measured using a previously described method [13] with slight modification of the substrate concentrations. The optimal substrate concentration for maximum reaction velocity was determined beforehand and employed in measuring specific activities. The assay mixture contained 50 mM Tris-HCl (pH 7.5), a reducing substrate (5–30 mM guaiacol, 20–100 mM 2,6-DMP, and 20–30 µM syringaldazine), and various concentrations of recombinant proteins and was preincubated at 30°C. In oxidation assays of rAtPrx25 activity, including the oxidation assay of C*c* ^2+^ described below, unexpectedly high calcium chloride was required to avoid deactivation, and 100 mM CaCl_2_ was added to the assay mixtures. The assay reaction was initiated by H_2_O_2_ addition to a final 1 mM concentration. C*c* ^2+^ was prepared by reducing C*c* ^3+^ with sodium hydrosulfite and the sodium hydrosulfite removed using a PD-10 column (GE Healthcare Bio-Sciences UK Ltd.). In a C*c* ^2+^ oxidation assay, the reaction mixture (500 µl) contained 30 µM C*c* ^2+^, various concentrations of recombinant proteins in the native or heat-denatured state (0–0.4 µM), and 0.1 mM H_2_O_2_ in 50 mM Tris-HCl buffer (pH 7.5) and preincubated at 30°C. The reaction was then carried out at 30°C. The progress of C*c* ^2+^ oxidation was monitored by spectral changes, evaluated in terms of decreased absorbance at 550 nm indicating oxidation of C*c* ^2+^ to C*c* ^3+^.

### Homology modeling

The three-dimensional structures of AtPrx-2, 25, and 71 were predicted with the homology-modeling server SWISS-MODEL (Swiss Institute of Bioinformatics, Lausanne, Switzerland; http://swissmodel.expasy.org/) [14]. The soybean peroxidase crystal structure [Protein Data Bank (PDB) entry 1FHF] was employed as the most appropriate template for AtPrx2, as it shares the highest amino acid sequence identity with soybean peroxidase among the known peroxidase crystal structures. In the same way, the ATP A2 crystal structure [PDB entry 1PA2] was selected as the most appropriate template for AtPrx-25 and 71. The obtained structures were visualized and analyzed with PyMol (an Open Source molecular viewing engine, DeLano Scientific LLC).

## Results

### Refolding and purification of recombinant proteins

**Figure 1:**
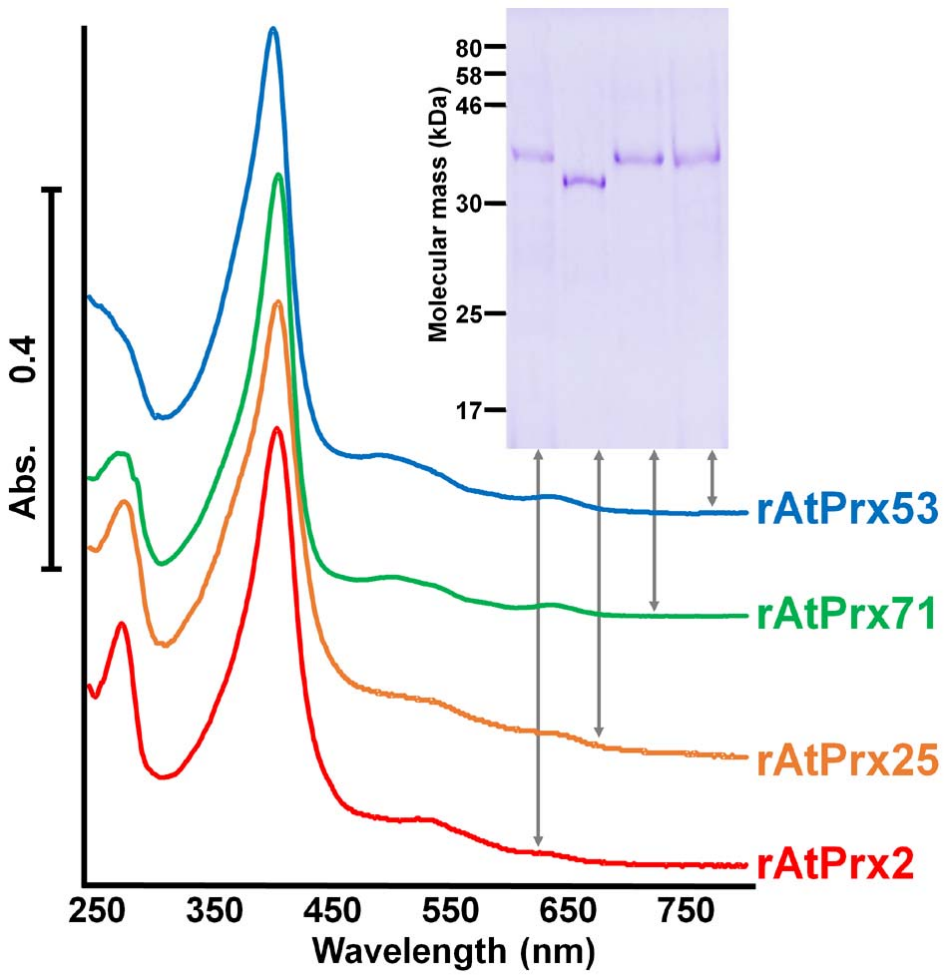
Absorption spectra of purified recombinant AtPrx2, AtPrx25, AtPrx53, and AtPrx71. Spectra from 250 to 800 nm of 10 µM proteins measured by UV-visible spectrometry and inset shows CBB staining of purified proteins (400 ng) after SDS–PAGE (12% gel) with molecular mass markers given in kDa on left.

Peroxidase activity was investigated using recombinant protein of each AtPrx, termed rAtPrx-2, 25, and 71, overexpressed in *E. coli*. Recombinant AtPrx53 was also produced as a comparison enzyme. As each recombinant protein from *E. coli* was recovered as an inclusion body lacking enzymatic activity, *in vitro*-refolding was applied to obtain the active renatured form. On the basis of known plant and fungal recombinant peroxidase refolding conditions [13], [15], [16], the compositions of refolding mixtures for rAtPrx-2, 25, and 53 were optimized by adjusting the concentrations of denaturizing agent (urea or guanidine), calcium chloride, hemin, oxidizing agent (GSSG or cystine), reducing agent (GSH or cystein), and protein as well as the pH and incubation time (Fig. S1). The refolding mixture for rAtPrx71 possessed sufficiently high peroxidase activity under the same refolding conditions as used for rCWPO-C such that further optimization was not needed. In the case of rAtPrx25, urea as the denaturizing agent resulted in misfolded-protein and cysteine/cysteine was found to be a better agent than GSSG/GSH for reconstructing the disulfide bond. Table 1 shows refolding conditions of inclusion body proteins after the optimization. The optimized refolding mixture of each recombinant protein, except for rAtPrx25, was purified by ion exchange chromatography on a MonoS or MonoQ column following dialysis in the appropriate buffer (see Materials and Methods). rAtPrx25 was purified by hydrophobic interaction chromatography on a HiPrep Butyl FF 16/10 column and gel filtration chromatography on a HiLoad 16/60 Superdex 75 column. After purification, the specific activities of rAtPrx-2, 25, 53, and 71 were increased to 108, 293, 47, and 2.8-fold, respectively, and the Reinheitszahl values (RZ) determined by the absorbance ratio A_400_/A_280_ were 1.8, 1.8, 2.8, and 2.7, respectively (Table 2). Purified rAtPrx fractions each presented a single band on CBB staining after SDS–PAGE (Fig. 1, inset), and their absorption spectrum exhibited a single Soret peak and two visible peaks (Fig. 1). An abundance of misfolded-protein resulted in an abnormally broad Soret peak (Fig. S2). Thus, as all purified enzyme preparations exhibited a spectrum specific to peroxidase, it was concluded that these recombinant proteins were effectively renatured.

**Table 2:** Purification of recombinant *Arabidopsis* peroxidases after refolding *in vitro*.

	Protein (mg)	Total activity (µmol/min)	Specific activity (µmol/min/mg protein)	Yield (%)	Purification (fold)	RZ (A400/A280)
rAtPrx2						
(1) Refolding mixture	5.0	59.6	11.9	100	1.0	0.59
(2) Dialysis	3.2	62.1	19.4	104	1.6	0.51
(3) Mono S	0.013	16.2	1291	27.2	108	1.8
rAtPrx25						
(1) Refolding mixture	30	27.6	0.920	100	1.0	0.81
(2) HiPrep Butyl FF	0.64	19.2	30.1	69.7	33	0.28
(3) Superdex75	0.031	8.4	270	30.3	293	1.8
rAtPrx53						
(1) Refolding mixture	14	96.6	7.07	100	1.0	0.61
(2) Dialysis	12	94.3	7.79	97.7	1.1	0.80
(3) Mono Q	0.058	19.4	333	20.1	47	2.8
rAtPrx71						
(1) Refolding mixture	0.65	218	337	100	1.0	0.60
(2) Dialysis	0.39	152	389	69.8	1.2	0.90
(3) Mono S	0.064	61.0	953	28.0	2.8	2.7

rAtPrx2 and rAtPrx71 activities determined by monitoring oxidation product of syringaldazine at 530 nm. rAtPrx25 and rAtPrx53 activities determined by monitoring oxidation product of guaiacol at 470 nm.

### Oxidation activity of recombinant proteins for monolignol model compounds

Because two methoxy groups of the syringyl unit cause severe steric hindrance compared with the guaiacyl unit, known plant peroxidases have difficulty oxidizing syringyl compounds, such as sinapyl alcohol. The oxidation activities of the recombinant enzymes were evaluated using three substrates, guaiacol, 2,6-dimethoxyphenol (2,6-DMP), and syringaldazine, because the oxidation product formation from these substrates could be monitored spectrophotometrically. Guaiacol, a representative of guaiacyl compounds, was used as a model for coniferyl alcohol, and 2,6-DMP and syringaldazine as models for sinapyl alcohol. The optimal substrate concentrations for maximal reaction velocity were determined in preliminary experiments. Table 3 shows each recombinant protein's specific activity for these substrates. rAtPrx53 showed higher specific activity for guaiacol than rAtPrx-2, 25, 53, and rCWPO-C, and the relative oxidation ratio for guaiacol/2,6-DMP/syringaldazine determined to be 1/0.12/0.026. This activity characteristic was quite similar to that of HRP-C. In contrast, rAtPrx-2, 25, and 71 showed higher specific activity for both syringyl compounds than rAtPrx53 and HRP-C. In particular, rAtPrx-2 and 71 preferred syringyl more than guaiacyl compounds as substrates, and the relative oxidation ratios were determined to be 1/2.1/15 and 1/3/3.5, respectively. Those properties were analogous to those of CWPO-C.

**Table 3:** Oxidation of guaiacol, 2,6-DMP, and syringaldazine by purified recombinant proteins and HRP-C.

Peroxidase	Purification process	Guaiacol				2,6-DMP				Syringaldazine			
rAtPrx2	Mono S	86	±	1.8	(1.0)	176	±	8.4	(2.1)	1291	±	84	(15)
rAtPrx25	Superdex 75	270	±	7.1	(1.0)	93	±	0.54	(0.34)	276	±	1.1	(1.0)
rAtPrx71	Mono S	270	±	4.3	(1.0)	805	±	19	(3.0)	953	±	3.5	(3.5)
rAtPrx53	Mono Q	333	±	0.45	(1.0)	39	±	0.80	(0.12)	8.6	±	0.20	(0.026)
rCWPO-C*	HiPrep Butyl FF16/10	120	±	2.4	(1.0)	480	±	29	(4.0)	1077	±	25	(8.9)
HRP-C*	Commercial Product	372	±	5.1	(1.0)	50	±	1.8	(0.14)	114	±	2.2	(0.31)

Oxidation activity (mean ±standard deviation, *n* = 3) expressed as µmol·min^−1^ per mg protein; relative activities in parentheses; relative activity of each protein determined by comparison with guaiacol activity, set to 1.0.

* Shigeto et al. 2012 [13].

### Oxidation of ferrocytochrome *c* by rAtPrx proteins

**Figure 2:**
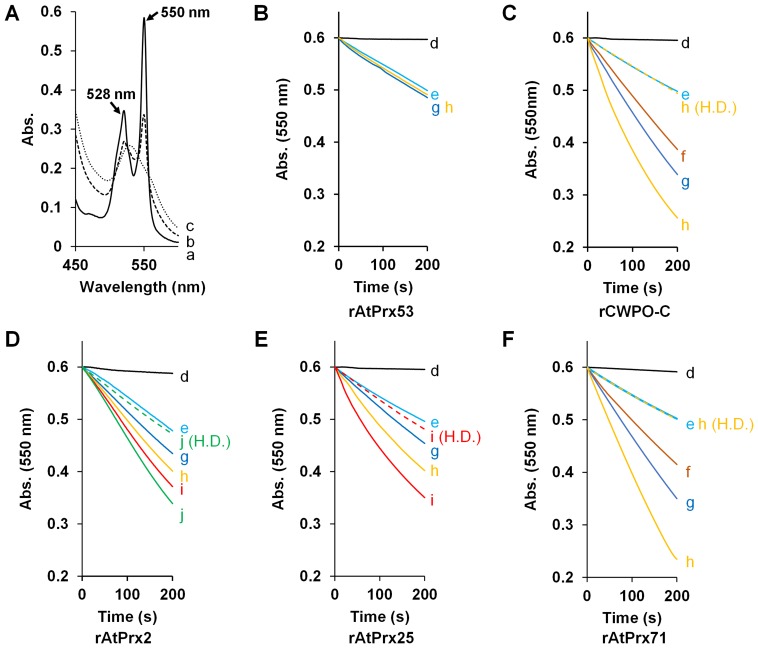
Spectrophotometric demonstration of C*c* ^2+^ oxidation. A: Spectral changes of C*c* ^2+^ during incubation with 0.1 µg rCWPO-C. Optical spectra between 450 and 600 nm recorded for C*c* ^2+^ in 50 mM Tris-HCl before (trace a) and after incubation with 0.1 mM H_2_O_2_ and 0.1 µM rCWPO-C for 180 sec (trace b) and 300 sec (trace c). B–F: Time course of C*c* ^2+^ oxidation by recombinant peroxidases. Absorbance monitoring started immediately after H_2_O_2_ addition; spontaneous C*c* ^2+^ oxidation monitored without H_2_O_2_ addition (trace d); protein concentrations: trace e, 0 µM; trace f, 0.05 µM; trace g, 0.1 µM; trace h, 0.2 µM; trace i, 0.3 µM; and trace j, 0.4 µM; complementary trace (H.D), absorption change with heat-denatured protein at given concentration.

The oxidation ability of these recombinant enzymes for large substrates was evaluated using ferrocytochrome *c* (C*c* ^2+^). Both C*c* ^2+^ and lignin polymer have too large molecular size to enter the plant peroxidase heme pocket. When C*c* ^2+^ is converted to C*c* ^3+^ by a single-electron oxidation, the absorption maximum at 550 nm decreases according to the degree of oxidation (Fig. 2A), and thus, C*c* ^2+^ oxidation is easily monitored as decreases in 550 nm absorption. The reaction was started by adding H_2_O_2_ to a reaction tube containing C*c* ^2+^ and the recombinant protein at different concentrations. No significant absorbance decreases were observed in reactions containing 0.1 and 0.2 µM rAtPrx53, which indicated that C*c* ^2+^ could not enter to the peroxidase heme pocket (Fig 2B). In contrast, 0.05, 0.1, and 0.2 µM rCWPO-C converted C*c* ^2+^ to C*c* ^3+^ and the observed oxidation velocities was clearly dependent on the protein concentration (Fig. 2C). rAtPrx-2, 25, and 71 also converted C*c* ^2+^ to C*c* ^3+^ (Fig. 2D, E, and F) at velocities dependent on protein concentration. The oxidation activities of rAtPrx-2, 25, and 71 for C*c* ^2+^ were estimated to be one-third, half, and equal to that of rCWPO-C, respectively.

### Prediction of the oxidation mechanism

**Figure 3:**
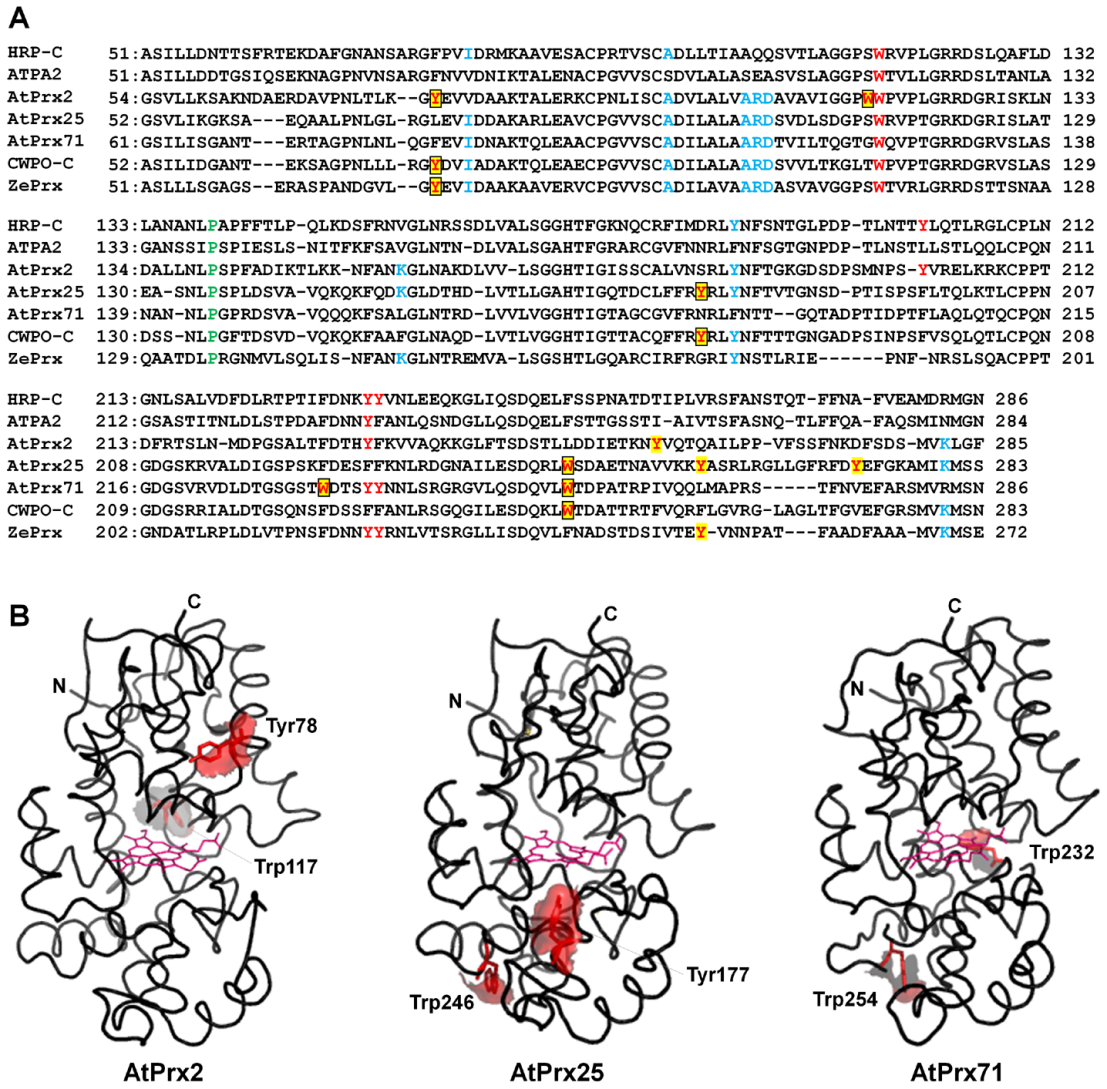
Primary and predicted 3-D structure of AtPrx2, AtPrx25, and AtPrx71. A: Amino acid alignment of plant peroxidases, HRP-C, AtPrx-53, 2, 25, 71, and ZePrx; conserved Pro139 in AtPrx53 in green structural motifs common to S peroxidases previously proposed (Ros Barceló et al. 2007, Novo-Uzal et al. 2013) in blue; tyrosine and tryptophan residues, presented in three peroxidases, AtPrx-2, 25, and 71, but not in AtPrx53 and HRP-C highlighted in yellow; possible oxidation sites enclosed in box. B: Predicted 3-D structures of AtPrx-2, 25, and 71 by homology-modeling performed with SWISS-MODEL using PDB entry (http://www.pdb.org/pdb/home/home.do) 1FHF (for AtPrx2) and 1PA2 structure (for AtPrx-25 and 71) as template; N and C-terminus of protein molecules labeled; and possible oxidation sites on protein surface in red.

The oxidation abilities of rAtPrx-2, 25, and 71 toward C*c* ^2+^ suggested that the substrate oxidation site existed on the proteins' surfaces, similar to Try74 and Tyr177 in CWPO-C. It has been reported that tryptophan can act as a redox active residue, similar to tyrosine, in fungal enzymes [17]–[19] and the Y74W CWPO-C variant [20]. Exposed tyrosine and tryptophan on protein surfaces can thus be considered as unique substrate oxidation sites and, specifically, the tyrosine and tryptophan residues present in these three peroxidases but not present in AtPrx53 and HRP-C were considered preferential candidates for oxidation sites (Fig. 3A, underlined residues). Following homology modeling of AtPrx-2, 25, and 71 by SWISS-MODEL was conducted to narrow the possible oxidation sites exposed on the proteins' surfaces. Tyr78 and Trp117 of AtPrx2 (Fig. 3B), Tyr177 and Trp246 of AtPrx25 (Fig. 3C), and Trp232 and Trp254 of AtPrx71 were on the shortlist for putative oxidation sites (Fig. 3D). Tyr78 of AtPrx2 and Tyr177 of AtPrx25 corresponded to the catalytic Try74 and Tyr177 in CWPO-C, respectively. Although AtPrx71 did not possess a similar tyrosine residue, a unique tryptophan residue at position 232 was located near the heme.

## Discussion

Oxidation of both growing lignin polymers and monolignols is indispensable for lignin biosynthesis. The molecular sizes of the lignin oligomers or polymers are too large to enter the heme-pocket active site of plant peroxidases [21]. Furthermore, as shown in Table S2, only a few peroxidases in the literatures can oxidize S unit. These facts posed a question for the progress of the lignin polymerization reaction mechanism. Based on modeling studies, AtPrx53 was concluded to have Pro139 overlapping the substrate-binding site such that a methoxy group in sinapyl alcohol hinders substrate docking [22]. Instead, it is suggested that a coniferyl alcohol radical acts as an intermediate in the oxidation of synapyl alcohol and lignin polymer [23]. On the other hand, a distinct characteristic of lignifying xylem peroxidases is their ability to oxidize syringyl compounds [24], and radical transfer from coniferyl alcohol to polymeric lignols occurred only slightly [10]. These observations imply that the lignin polymerization mechanism could not be explained sufficiently by a radical transfer system *via* a mediator. In the last ten-odd years, three plant peroxidases, ZePrx from *Zinnia elegans* [25], BPX1 from *Betula pendula* [26], and CWPO-C from *Populus alba* L. [9], have been reported to have higher oxidation activities toward sinapyl than coniferyl alcohol. Notably, CWPO-C exhibits higher oxidation activity for sinapyl alcohol by approximately ten times that of HRP-C, is able to oxidize C*c* ^2+^, and synthesizes lignin polymer *in vitro* [10]. However, the oxidation ability of plant peroxidase catalyzing lignin polymerization *in vivo* remains unclear.

**Figure 4:**
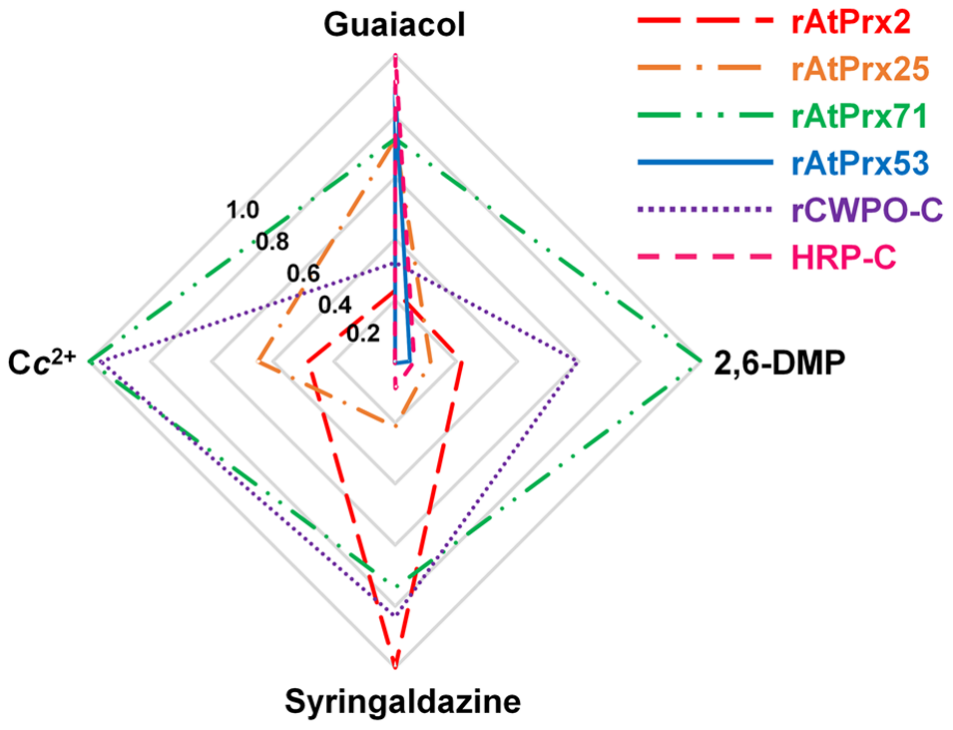
A cobweb chart illustration of the relative oxidation activities of recombinant peroxidases and native HRP-C. Peroxidase activities listed in Table 3 converted to relative values calculated with the highest activity among six peroxidases set to 1.0; C*c* ^2+^ oxidation activity estimated by decreased C*c* ^2+^ concentration listed in Figure 3, except for HRP-C; and C*c* ^2+^ oxidation by HRP-C previously reported (Sasaki et al. 2004).

In this study, the oxidation activities of three genus *Arabidopsis* plant peroxidases, AtPrx-2, 25, and 71, formerly confirmed to be involved in lignification, were evaluated using monolignol model compounds and C*c* ^2+^, the latter representing an oxidative property of the lignin polymer. As expected, rAtPrx53 and HRP-C exhibited substantially lower specific activities for the two syringyl compounds than for guaiacol and also did not oxidize C*c* ^2+^. In contrast, rAtPrx-2, 25, and 71 readily oxidized the same two syringyl compounds as well as C*c* ^2+^; the summarized oxidation results are depicted in a cobweb chart (Fig. 4). The oxidation abilities for both syringyl compounds and C*c* ^2+^ were conserved in all three AtPrx enzymes. This observation reflected that AtPrx-2, 25, and 71 can oxidize these substrates without the steric hindrance that restricts substrates from entering their heme pocket active site. In other words, these enzymes have oxidation site(s) exposed or available on their protein surfaces, and it is highly likely that their oxidation activities toward C*c* ^2+^ as well as their activity toward the monolignol model compounds were dependent on these site(s). rAtPrx-2 and 71 showed higher oxidation activities for two syringyl compounds than for guaiacol, a property shared with CWPO-C which cannot oxidize guaiacol or syringyl compounds in its heme pocket [13]. This suggested that guaiacol and syringyl compound oxidation of rAtPrx-2 and 71 took place largely on the protein surfaces for the following two reasons. First, 2,6-DMP and syringaldazine were more easily oxidized than guaiacol because of their redox potential under steric hindrance-free conditions. The methoxy group's electron-donating property has been reported to reduce the redox potential of phenolic compounds, and guaiacol has a higher redox potential than 2,6-DMP and syringaldazine [27]. This situation is true for sinapyl alcohol, which has a lower redox potential than coniferyl alcohol [28]. And second, AtPrx-2 and 71 would not have large enough heme pockets to easily accept syringyl compounds. Their heme pocket entrances, estimated by homology modeling, are not larger than those of AtPrx53 and HRP-C (Fig. S3) and Pro139 is conserved as well, as it has been in AtPrx53 (Fig. 3A). AtPrx25 possessed intermediate oxidation characteristics between those of AtPrx53 and CWPO-C, and did not possess a sufficiently large heme pocket entrance, and conserves the mentioned proline. Considering the oxidation activity and predicted 3D-structure of AtPrx25, most of its oxidation activity for guaiacol might have originated from the heme pocket, and the oxidation activity on the protein surface toward monolignol model compounds would be lower than in CWPO-C, AtPrx-2, and 71. An oxidation reaction caused by an exposed protein radical will depend on the redox potential and not follow the presumed enzyme kinetics of a two-step reaction (step one, the substrate binds to the enzyme and step two, the substrate is converted to product and released). Therefore, to explain these enzymes' oxidation activities for monolignol model compounds, specific activity (µmol·min^−1^ per mg protein) was employed instead of steady-state kinetic constant values (*K*_m_, *k*_cat_, and turnover rate).

Poplar CWPO-C is the only peroxidase that has been shown to have exposed catalytic sites in plant peroxidases [13]. CWPO-C uses exposed Tyr74 and Tyr177 as the oxidation site, and conserved tyrosines, Tyr78 of AtPrx2 and Tyr177 of AtPrx25, were considered here as the most likely oxidation sites. Although AtPrx71 did not have an exposed tyrosine, unique Trp232 and Trp254 exposed on the protein surface might play a similar role. Exposed catalytic sites have been identified in some fungal peroxidases, including versatile peroxidase with catalytic Trp164 [18], [19], *Phanerochate chrysosporium* lignin peroxidase with catalytic Trp171 [17], and *Trametes cervina* lignin peroxidase with catalytic Tyr181 [29]. Long-range electron transfer (LRET) pathways from the exposed catalytic site to the buried heme cofactor were suggested by crystallographic studies of these peroxidases [17], [29], [30]. There may be a LRET pathway in CWPO-C, AtPrx-2, 25 and 71 similar to versatile peroxidase and lignin peroxidases, though crystallographic study is necessary to discuss detailed LRET pathway in CWPO-C, AtPrx-2, 25 and 71.

Plant peroxidases able to oxidize guaiacyl moieties but unable to oxidize efficiently syringyl moieties are called G peroxidase. Unlike G peroxidase, plant peroxidases able to oxidize both guaiacyl and syringyl moieties have been called S peroxidase [8] so far. It was reported that CWPO-C has not only a property of S peroxidase, but also has the oxidation ability for large molecule [10]. Therefore, CWPO-C should be included in new category of "all-round peroxidase". From the similar point of view, AtPrx-2, 25, and 71 are classified to the all-round peroxidase. The structural motifs of S peroxidases, which are amino acid sequences conserved among S peroxidases but not in G peroxidases, were proposed by Ros Barceló et al. (2007) [8] and Novo-Uzal et al. (2013) [31]. AtPrx-2, 25, and 71 conserve, indeed, all or part of these motifs (Fig. 3A). However, HRP-C, a representative G peroxidase, conserves part of these motifs and most AtPrxs (68 out of 73) share at least two of these motifs, indicating that it is difficult to search for S peroxidases using only these motifs. When it is presumed that S peroxidases oxidize syringyl compounds by a protein surface oxidation site, as in CWPO-C, it becomes a discriminating criterion regarding the peroxidase possessing exposed tyrosine and tryptophan, which form the substrate oxidation site, to distinguish G from S peroxidases. ZePrx, a typical S peroxidase, has Tyr73 that corresponds to Tyr74 in CWPO-C, and thus ZePrx might be likely to use the tyrosine as an oxidation site and able to oxidize not only syringyl compounds but larger substrates as well. It means that ZePrx may also be all-round peroxidase. Recently, AtPrx72, a homolog of ZePrx, has also been shown to be involved in *Arabidopsis* stem lignification by analysis of *Arabidopsis* knockout mutants [32]. The predicted mature AtPrx72 protein shows some exposed tyrosines. Such oxidation characteristics in plant peroxidases involved in lignification with *in vivo* evidence, including AtPrx72, are of interest and will provide additional knowledge regarding the mechanism of plant peroxidase-mediated lignin polymerization.

In conclusion, recombinant proteins of three plant peroxidases, AtPrx-2, 25, and 71, responsible for lignin polymerization in the *Arabidopsis* stem were produced, and their oxidation characteristics were examined. The results indicated that these peroxidases oxidized the large molecules required for direct oxidation of sinapyl alcohol and lignin polymers, unlike most known plant peroxidases. This characteristic clearly explained the lignin polymerization mechanism. Taking their oxidation activities and estimated structure into consideration, it was suggested that AtPrx-2, 25, and 71 have oxidation site(s) on their protein surfaces.

## Supporting Information

Figure S1 Parameter optimization for the *in vitro* refolding of recombinant AtPrx proteins. Denaturizing agent concentration (A), hemin concentration (B), pH (C), CaCl_2_ concentration (D), oxidizing agent concentration (E), Reducing agent concentration (F), Protein concentration (G), and incubation time (H) were systematically varied, and shown as relative activity of maximal yields of each. The basic conditions were: 48 h incubation at 4°C in 50 mM Tris–HCl buffer (pH 9.5) containing 3.25 M urea, 10 µM hemin, 40 mM CaCl_2_, 0.7 mM GSSG, 0.21 mM GSH, 0.2 mg/ml protein. Refolding efficiency was estimated by guaicol oxidation.
(TIF)

Figure S2 A normal and abnormal spectra of purified recombinant AtPrx25. To renature inactive recombinant AtPrx25 protein, a final concentration of 1 M urea or 0.25 M guanidine was used as denaturizing agent in refolding mixture; after purification (see Materials and methods), fraction with highest specific activity collected; and absorption spectrum measured by UV-visible spectrometry.
(TIF)

Figure S3 The entrance to the heme pocket of AtPrx proteins, HRP-C, and CWPO-C. Structure of AtPrx53 and HRP-C, and predicted structure of AtPrx2, 25, and 71 were as described in Materials and methods and Fig. 3 legend; predicted CWPO-C structure prepared as previously described [13]; and intensity of red deepness, level of hydrophobicity.
(TIF)

Table S1 Sequence of primers used to amplify cDNA sequences.
(DOCX)

Table S2 Oxidation property of plant peroxidases for G and S units.
(DOCX)
