# Supplementary figures and images for: Catalytic Profile of Arabidopsis Peroxidases, AtPrx-2, 25 and 71, Contributing to Stem Lignification
Source: PLoS One. 2014 Aug 19;9(8):e105332. doi: 10.1371/journal.pone.0105332 (PMC4138150; doi:10.1371/journal.pone.0105332)

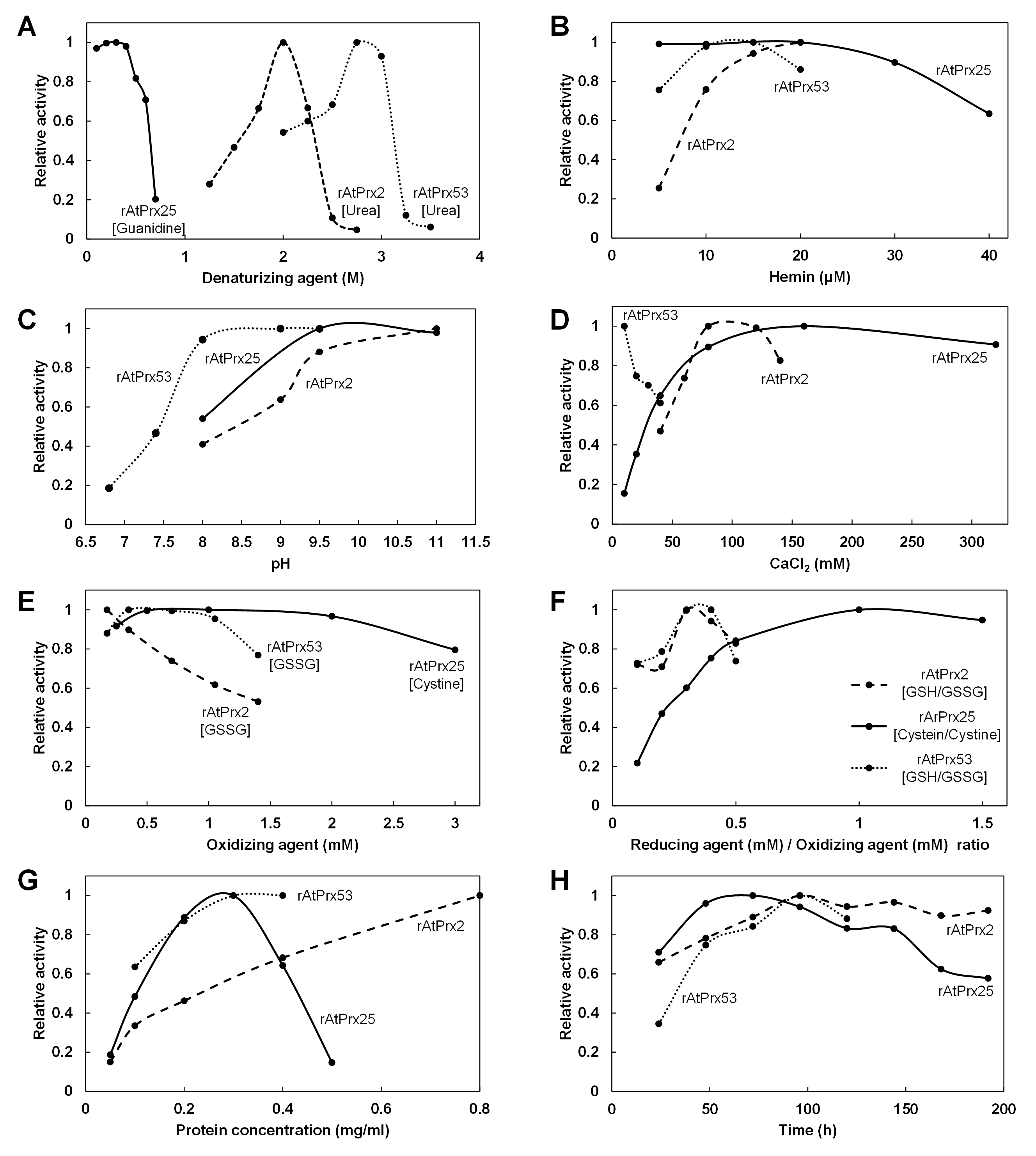

Supplement: Figure S1 — Parameter optimization for the in vitro refolding of recombinant AtPrx proteins. Denaturizing agent concentration (A), hemin concentration (B), pH (C), CaCl2 concentration (D), oxidizing agent concentration (E), Reducing agent concentration (F), Protein concentration (G), and incubation time (H) were systematically varied, and shown as relative activity of maximal yields of each. The basic conditions were: 48 h incubation at 4°C in 50 mM Tris–HCl buffer (pH 9.5) containing 3.25 M urea, 10 µM hemin, 40 mM CaCl2, 0.7 mM GSSG, 0.21 mM GSH, 0.2 mg/ml protein. Refolding efficiency was estimated by guaicol oxidation. (TIF) [file pone.0105332.s001.tif]

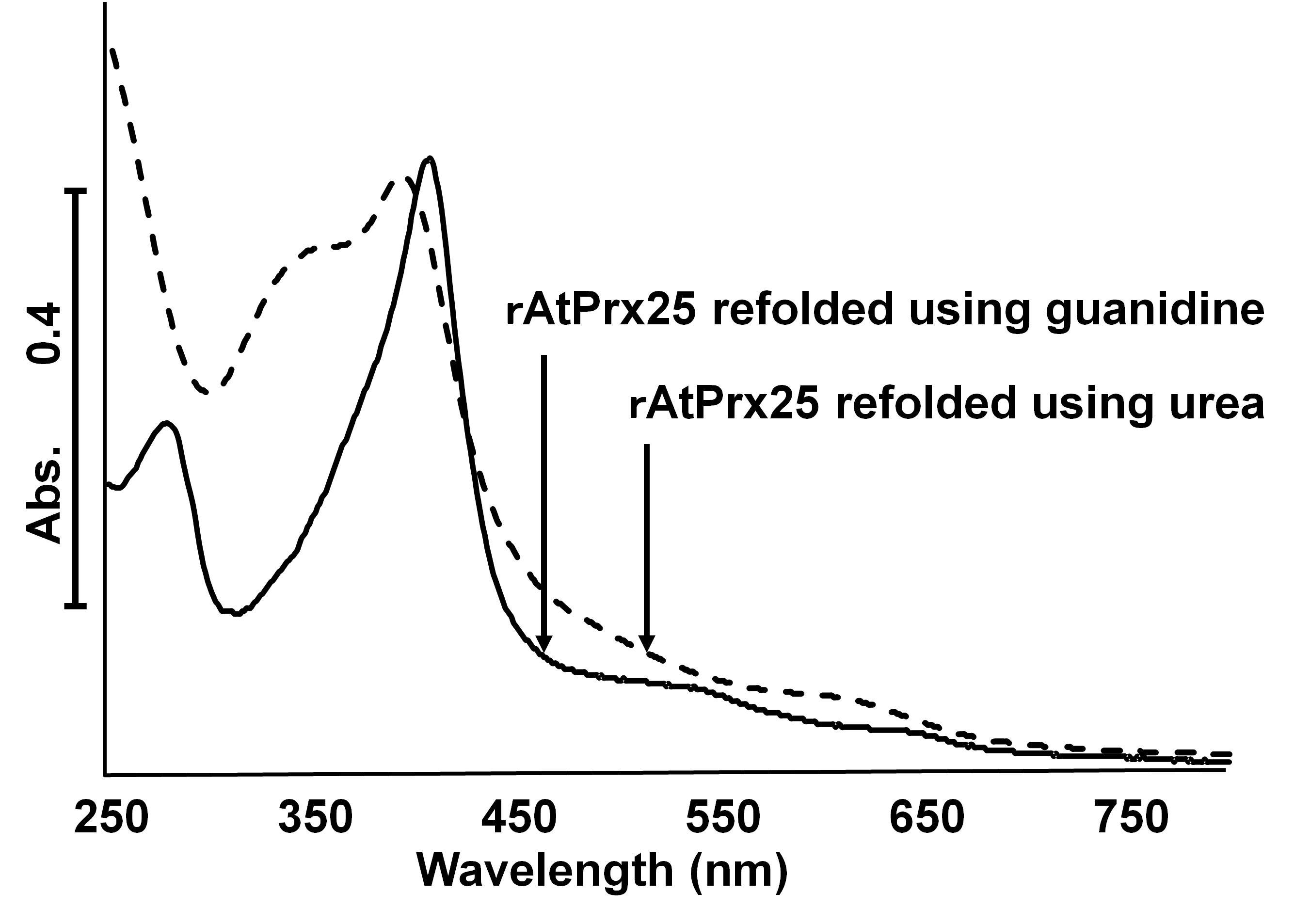

Supplement: Figure S2 — A normal and abnormal spectra of purified recombinant AtPrx25. To renature inactive recombinant AtPrx25 protein, a final concentration of 1 M urea or 0.25 M guanidine was used as denaturizing agent in refolding mixture; after purification (see Materials and methods), fraction with highest specific activity collected; and absorption spectrum measured by UV-visible spectrometry. (TIF) [file pone.0105332.s002.tif]

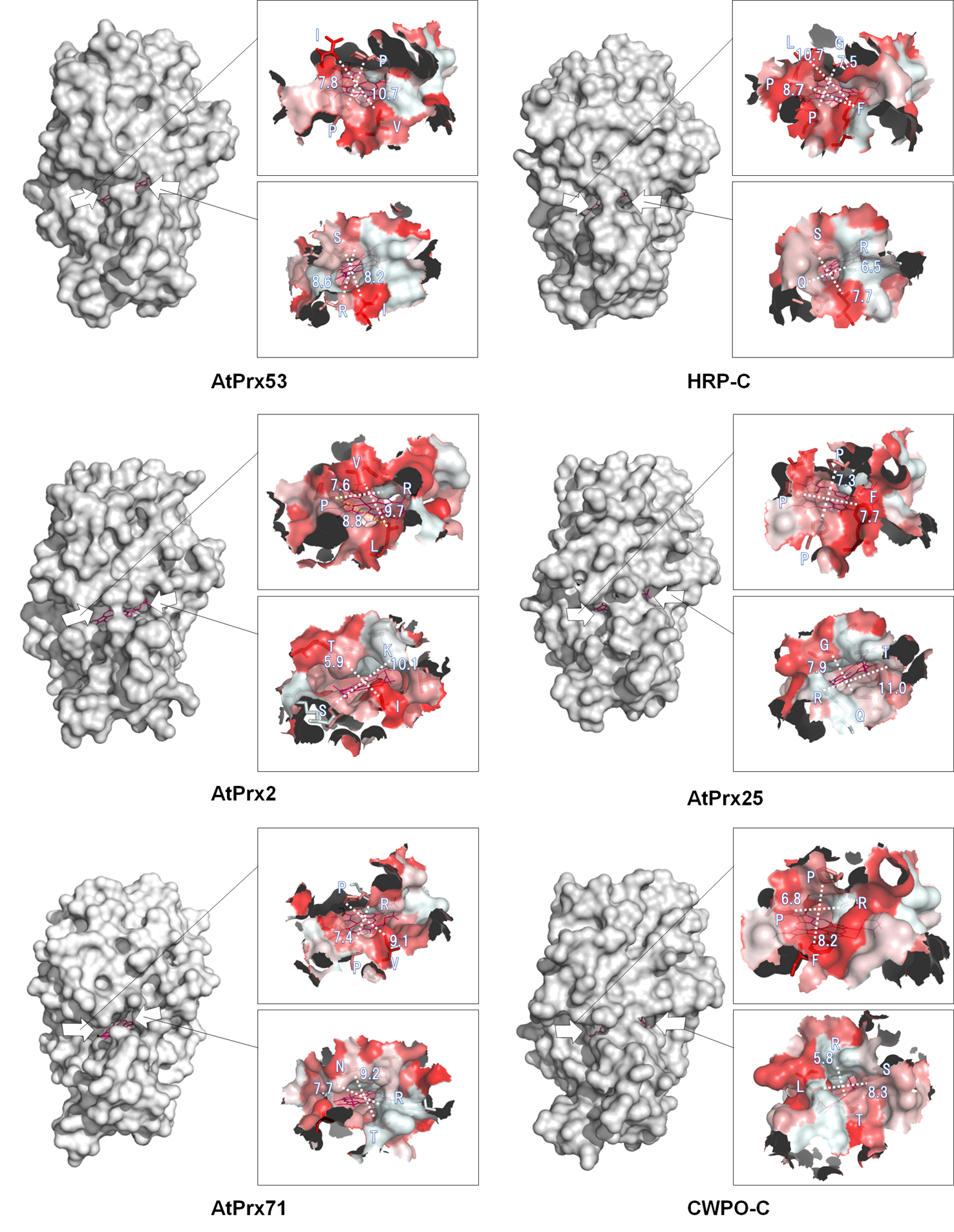

Supplement: Figure S3 — The entrance to the heme pocket of AtPrx proteins, HRP-C, and CWPO-C. Structure of AtPrx53 and HRP-C, and predicted structure of AtPrx2, 25, and 71 were as described in Materials and methods and Fig. 3 legend; predicted CWPO-C structure prepared as previously described [13]; and intensity of red deepness, level of hydrophobicity. (TIF) [file pone.0105332.s003.tif]
