# Supplementary material for: Catalytic Profile of Arabidopsis Peroxidases, AtPrx-2, 25 and 71, Contributing to Stem Lignification
Source: PLoS One. 2014 Aug 19;9(8):e105332. doi: 10.1371/journal.pone.0105332 (PMC4138150; doi:10.1371/journal.pone.0105332)
Supplement: Table S1 — Sequence of primers used to amplify cDNA sequences. (DOCX) [file pone.0105332.s004.docx]

**Table S1. Sequence of primers used to amplify cDNA sequences.**

Gene Forward Primer Reverse Primer

*AtPrx2* 5’-attagcggatccattccacagttgcttgacctcg-3’ 　 5’-tatgcgagctcttagttagggaaggcgcatctc-3’

*AtPrx25* 5’-cacgcggatcccagttgttaaaaaatggttattattcaac-3’ 5’ -aagatagagctcctagttcaccttggaacaaac-3’

*AtPrx53* 5’-actattggatccgcgcaactaaacgcaacattttactc-3’ 5’-tatgcgagctctcaacttccattaaccttcttacagtc-3’

*AtPrx71* 5’-attattggatcccaagccaccgcaaggcctgg-3’ 　 5’-tgtagcgagctcttaattaaccgcagagcaaaccctacgg-3’
