# Supplementary material for: Catalytic Profile of Arabidopsis Peroxidases, AtPrx-2, 25 and 71, Contributing to Stem Lignification
Source: PLoS One. 2014 Aug 19;9(8):e105332. doi: 10.1371/journal.pone.0105332 (PMC4138150; doi:10.1371/journal.pone.0105332)
Supplement: Table S2 — Oxidation property of plant peroxidases for G and S units. (DOCX) [file pone.0105332.s005.docx]

**Table S2. Oxidation property of plant peroxidases for G and S units.**

| Peroxidase | Spieces | Evaluation | Substrate | | S/G | Reference |
| --- | --- | --- | --- | --- | --- | --- |
|  |  |  | Coniferyl alcohol | Sinapyl alcohol |  |  |
| Acer peroxidase | *Acer pseudoplatanus* | specific activity (µmol min^-1^ mg protein^-1^) | 0.17 | 0.08 | 0.47 | Sterjiades et al. 1993 |
| anionic peroxidase | *Solanum tuberosum* | Vmax (pkat) | 174.6 | 12.5 | 0.07 | Bernards et al. 1999 |
| P1 | *Zinnia elegans* | specific activity (μmol min^–1^ U^–1^*) | 10.1 | 0 | 0.00 | Sato et al. 1999 |
| P3 | *Zinnia elegans* | specific activity (μmol min^–1^ U^–1^*) | 13.3 | 3 | 0.23 | Sato et al. 1999 |
| P5A | *Zinnia elegans* | specific activity (μmol min^–1^ U^–1^*) | 12.7 | 0 | 0.00 | Sato et al. 1999 |
| P5B | *Zinnia elegans* | specific activity (μmol min^–1^ U^–1^*) | 6 | 0 | 0.00 | Sato et al. 1999 |
| CWPO-C | *Populus alba* | specific activity (µmol min^-1^ mg protein^-1^) | 433 | 705 | 1.63 | Aoyama et al. 2002 |
| CWPO-A | *Populus alba* | specific activity (µmol min^-1^ mg protein^-1^) | 1572 | 38 | 0.02 | Aoyama et al. 2002 |
| HRP-C | *Armoracia rusticana* | specific activity (µmol min^-1^ mg protein^-1^) | 1526 | 40 | 0.03 | Aoyama et al. 2002 |
| rAoPOX1 | *Asparagus asparagoides* | Vmax (mM s^-1^) | 0.406 | 0.111 | 0.27 | Takeda et al. 2003 |
| ZePrx34.70 (33.44) | *Zinnia elegans* | k*_cat_* (s^-1^) | 149 (150) | 331 (397) | 2.22 (2.65) | Gabaldón et al. 2005 |
| PAPX4 | *Picea abies* | specific activity (nkat mg protein^– 1^) | 2470 | N.D. | 0.00 | Koutaniemi et al 2005 |
| PAPX5 | *Picea abies* | specific activity (nkat mg protein^– 1^) | 2290 | N.D. | 0.00 | Koutaniemi et al 2005 |
| ZPO-C | *Zinnia elegans* | specific activity (μmol min^–1^ U^–1^*) | 11.4 | 2.31 | 0.20 | Sato et al. 2006 |
| BPX1 | *Betula pendula* | specific activity (nkat mg protein^– 1^) | 18.8 | 51.8 | 2.76 | Marjamaa et al. 2006 |
| BPX2 | *Betula pendula* | specific activity (nkat mg protein^– 1^) | 4453.7 | 199.2 | 0.04 | Marjamaa et al. 2006 |
| BPX3 | *Betula pendula* | specific activity (nkat mg protein^– 1^) | 46.7 | 26.5 | 0.57 | Marjamaa et al. 2006 |
| PPX1 | *Picea abies* | specific activity (nkat mg protein^– 1^) | 137.5 | 23.9 | 0.17 | Marjamaa et al. 2006 |
| PPX2 | *Picea abies* | specific activity (nkat mg protein^– 1^) | 1.7 | 1.3 | 0.76 | Marjamaa et al. 2006 |
| PPX3 | *Picea abies* | specific activity (nkat mg protein^– 1^) | 22.3 | 2.6 | 0.12 | Marjamaa et al. 2006 |
| PPX4 | *Picea abies* | specific activity (nkat mg protein^– 1^) | 1.9 | 0.4 | 0.21 | Marjamaa et al. 2006 |
| PPX5 | *Picea abies* | specific activity (nkat mg protein^– 1^) | 27.3 | 5.7 | 0.21 | Marjamaa et al. 2006 |
| GbPrx32.49 | *Ginkgo biloba* | k*_cat_* (s^-1^) | 40.3 | 0.4 | 0.01 | Novo Uzal et al. 2009 |
| SmaPrx2 | *Selaginella martensii* | specific activity (nkat µg protein^-1^) | 3.6 | 0.78 | 0.22 | Martínez-Cortés et al. 2012 |
| SmaPrx3 | *Selaginella martensii* | specific activity (nkat µg protein^-1^) | 19 | 1.94 | 0.10 | Martínez-Cortés et al. 2012 |
|  |  |  | Coumaric acid | Sinapinic acid |  |  |
| HRP A2 | *Armoracia rusticana* | k*_cat_* (s^-1^) | 165 | 4 | 0.02 | Nielsen et al. 2001 |
| HRP-C | *Armoracia rusticana* | k*_cat_* (s^-1^) | 1522 | 3 | 0.00 | Nielsen et al. 2001 |
| AtPrx53 | *Arabidopsis thaliana* | k*_cat_* (s^-1^) | 48 | 1.3 | 0.03 | Nielsen et al. 2001 |

＊One unit of peroxidase oxidizes 1 μmol of guaiacol min^–1^.　N.D., not determined due to lack of activity
